# Impact of REM Sleep Behavior and Sleep Talking on Mortality in Parkinson's Disease

**DOI:** 10.7759/cureus.52565

**Published:** 2024-01-19

**Authors:** Eemil Partinen, Ari Ylikoski, Mariusz Sieminski, Markku Partinen

**Affiliations:** 1 Department of Neurology, University of Helsinki, Helsinki, FIN; 2 Helsinki Sleep Clinic, Terveystalo Healthcare, Helsinki, FIN; 3 Department of Neurology, Social Insurance Institution of Finland (KELA), Helsinki, FIN; 4 Department of Emergency Medicine, University of Gdansk, Gdansk, POL; 5 Helsinki Sleep Clinic, Vitalmed Research Center, Helsinki, FIN; 6 Department of Clinical Neurosciences, University of Helsinki, Helsinki, FIN

**Keywords:** mortality, rbd, rem sleep behaviour disorder, parkinson’s disease, sleep disorders, parasomnias, sleep talking

## Abstract

Background

REM sleep behavior disorder (RBD) is a prodromal marker for Parkinson’s disease (PD) and other alpha-synucleinopathies. Sleep talking (ST) is an isolated symptom and is frequent in PD and RBD. Here, we investigate the associations of ST and RBD with the mortality of PD patients.

Patients and methods

A total of 1,500 PD patients were randomly selected from the registry of the Finnish Parkinson’s Association. Of the 855 that participated at baseline, 645 gave permission for follow-up studies. We gathered a completely filled sleep questionnaire and mortality information from 384 subjects. The Nelson-Aalen test and Cox hazard ratios (HR) were used for mortality analyses.

Results

The mean follow-up time was 4.3 years (0.3-7.0). PD patients with RBD or frequent ST had more non-motor symptoms. Depression, hallucinations, constipation, and excessive daytime sleepiness were more prevalent among subjects with RBD. Subjects with RBD and frequent ST (talking in their sleep ≥ once per week) had increased mortality (HR: 1.90, 95% CI: 1.18-3.06). RBD without frequent ST was not associated with mortality (HR: 0.77, 95% CI: 0.4-1.5). Frequent ST was associated with increased mortality when adjusted for age, PD duration, depression, gender, RBD, BMI, and hallucinations (HR: 2.22, 95% CI: 1.10-4.51). Additionally, age, duration of PD, arterial hypertension, and lower BMI were associated with increased mortality. Male gender, dopaminergic medication, depression, and hallucinations were not significantly associated with mortality.

Conclusions

RBD with frequent ST and ST alone appear to be risk factors for mortality in PD. Frequent ST may be a sign representing wider neurodegeneration. RBD subjects and frequent sleep talkers demonstrated more non-motor symptoms compared to PD without RBD or ST. Our findings have clinical implications. It remains to be seen if frequent ST indicates a poorer prognosis. Prospective studies are needed to find whether frequent ST is also a risk factor for developing PD.

## Introduction

Parkinson’s disease (PD) is a severe progressive neurodegenerative disease. PD symptoms are divided into motor and non-motor symptoms (e.g., sleep symptoms). Known factors that increase mortality in PD include increasing age, presence of dementia, male sex, presence of psychotic symptoms, and depression [1]. PD patients with dementia have a particularly high risk of mortality [2,3]. The mechanisms behind these risk factors are not well-known. 

Information on the role of sleep disorders related to mortality in PD is scarce. We have found only one earlier report from Japan indicating that sleep talking (ST) differentiates dementia with synucleinopathy from other types of dementias [4]. An abundance of evidence points to REM sleep behavior disorder to be a risk factor for the development of PD and other alpha-synucleinopathies. Several reports also show that RBD is associated with more severe motor and non-motor PD manifestations, including cognitive decline [5]. However, it remains unclear whether RBD is a risk factor for mortality in PD.

In a previous study, ST has been strongly associated with RBD [6]. The prevalence of ST in PD patients without RBD is 5.6%, but this rises to 47.7% if RBD is present [6]. Occasional ST is a normal phenomenon. Less is known about frequent ST (at least three nights per week). The role of frequent ST in PD has not been studied before. In this study, we examined whether frequent ST is a risk factor for mortality.

## Materials and methods

Participant selection

In 2010, 1,500 Parkinson's patients were randomly selected from the registry of the Finnish Parkinson's Association. Of these, 53 had already deceased, were hospitalized, were unable to answer, were relatives, or did not have PD. A structured questionnaire was sent to 1,447 eligible patients with PD. The questionnaire and study population are described in earlier publications [6]. All patients who returned the questionnaire and had been diagnosed with PD were included in the study.

Data collection

The questionnaire had 207 items, including items from the Basic Nordic Sleep Questionnaire (BNSQ) [7]. The BNSQ is a validated questionnaire about sleep and sleep disorders consisting of 21 items. Other items included in the questionnaire were demographic information, time of PD diagnosis, and onset of symptoms. In this study, the duration of PD was calculated from the time of diagnosis. BMI was calculated from self-reported height and weight. Smoking and alcohol consumption were reported as doses per week (one dose = one cigarette, or 12 g of alcohol). The presence of arterial hypertension and diabetes were asked separately. The questionnaire did not specify whether a patient had type 1 or type 2 diabetes. Patients reported the name, dose, and dosing schedule of currently used medications. Total daily levodopa equivalent doses (LED) were calculated using separate conversion factors for different medications [8].

The Marburg questionnaire (RBDSQ) was used [9]. The RBDSQ is perhaps the most commonly used questionnaire for screening for RBD. Cronbach's alpha is 0.73 [10]. We used a limit of ≥6 for RBD. The presence of ST was screened by the question: “How often during the last year have you been talking in your sleep?”. The responders were instructed to ask other people if they did not know about their ST history. The response alternatives were from the BNSQ: 1) “never”; 2) “less than once a month”; 3) “less than once a week”; 4) “once or twice a week”; 5) “3 to 5 days a week”; and 6) “every night” [7]. Subjects were considered "sleep talkers” if they reported ST at least once a week. ST less than once per week and other sounds during sleep (i.e., laughing, screaming, and mumbling) were regarded as sleep vocalization. Depression was assessed using the World Health Organization WHO5 well-being scale WHO5 (score 0-100), which has been validated for screening depression in a population of PD patients with Cronbach's alpha of 0.83. The cutoff point for depression is ≤ 50 [11]. We chose the WHO5 scale because, unlike most depression scales, it does not include any questions about sleep, as we have noted that positive answers reflecting poor sleep could contribute to false-positive depression scores in subjects with insomnia or other sleep disorders.

Different hallucinations were asked using the same response alternatives from 1 to 6. The questionnaire included separate questions on hypnagogic, hypnopompic, nighttime, and evening-time hallucinations. If a patient had any type of hallucinations once a week or more, hallucinations were regarded as present. The presence of constipation was regarded as having hard bowel movements three or more times per week. The subjects were considered to have olfactory dysfunction if they estimated that their olfactory function (sense of smell) was worse than that of other people or that they could not smell anything (anosmia). Daytime sleepiness was assessed using the Epworth Sleepiness Scale (ESS).

Statistical analysis

Variables for mortality models were selected based on previous known predictors, possible confounding variables, and clinical expertise. The time variable was set to days from the date of answering the first questionnaire to the latest known timepoint (returning the second questionnaire, last known contact, or confirmed time of death). The Nelson-Aalen test and Cox hazard ratios (HR) were used for mortality analyses. Age and gender were included in models as known major demographic variables. Models were built using forward and backward stepwise methods if the removal of the variable did not significantly worsen the model. Model 1 was selected using a P < 0.1 as the cutoff point and including other major variables.

Descriptive results of continuous variables are given as means and 95% confidence intervals (CI; also median and interquartile ranges are given for non-normally distributed variables). The Shapiro-Wilk test was used to test for the normality of the distributions. The Mann-Whitney U-test was used for nonparametric variables and the Student’s t-test for parametric variables. Categorical values were expressed in numbers and percentages and analyzed by Pearson's chi-square and Fisher's exact tests. P-value < 0.05 was considered statistically significant. All analyses were performed using Stata 17.0 (StataCorp LLC, USA).

This study was approved by the Regional Ethics Review Board of the Helsinki and Uusimaa Hospital District (HUS). The study was conducted according to the Declaration of Helsinki.

## Results

The response rate was 59% from 1,447 patients to whom the questionnaire was sent (original cohort, N=855), and of these, 81% returned a fully or an almost fully answered questionnaire (N=689). A follow-up questionnaire was sent to all patients of the original cohort who had responded to the questionnaire and who had given written consent for further contacts (N=645). Thirty-nine patients were lost to follow-up.

We were able to collect follow-up information from 384 patients (60%) who had returned fully answered questionnaires. These (N=384) formed our study cohort. The mean follow-up time was 4.3 years, ranging from 0.3 to 7.0 years. Altogether, 293 subjects were alive at the time of follow-up, 82 had died, and six were living in elderly homes. We could not specify whether the three subjects were living at home or in an elderly home. We tested for differences between the study cohort (N=384) and the original cohort N =855). At the moment of the first survey (time of formation of the original cohort), the mean age was 68.9 years (95% CI: 68.4-69.5). The mean age of our study cohort was 67.4 years (66.5-68.3; P=0.002). The gender distribution and prevalence of ST and RDB were similar between the populations. The proportion of men was 53% in the original cohort and 56% in our study cohort. In the original cohort, the prevalence of frequent ST was 23.3% (95% CI: 20.3-26.4), and the prevalence of RBD was 32.5% (95% CI: 29.3-35.9).

Characteristics of the subjects in the cohort at baseline are provided in Table 1. The difference between subjects with and without RBD and subjects with RBD together with and without frequent ST is also presented in Table 1. Subjects with RBD differed from non-RBD subjects in many ways (see Table 1). RBD subjects were older. Non-motor symptoms were more prevalent among RBD subjects, together with a higher intake of PD medications and a higher ESS score. RBD patients with ST did not differ from RBD patients without ST based on non-motor symptoms, duration of PD, or intake of PD medications. Arterial hypertension was more prevalent among subjects with RBD without ST.

**Table 1:** Demographics: Comparison between RBD and non-RBD subjects together with RBD subjects with sleep talking (ST) and without sleep talking Values are given as means or percentages, followed by a 95% confidence interval in parentheses. The P-value is calculated for the difference between RBD vs without RBD and ST vs without ST. Statistically significant differences are printed in bold. NS: Statistically not significant; BMI: Body mass index; PD: Parkinson’s disease; LED: Levodopa equivalent dose; RBDSQ: REM sleep behavior disorder screening questionnaire; ESS: Epworth Sleepiness Scale

	All N=384	All with RBD N=126	All wo RBD N=258	P-value	RBD with ST (N=70) % or mean (95% CI)	RBD wo ST (N=56)	P-value	P-value RBD+ST vs all
Age (years)	67.4 (66.5-68.3)	69.1 (67.6-70.6)	66.6 (65.5-67.7)	0.005	68.3 (66.1-70.5)	70.1 (68.1-72.2)	NS	NS
Age > 65 years	60.4% (55.5-65.3)	68.3% (60.0-76.5)	56.6% (50.5-62.7))	0.03	61.4% (49.7-73.1)	76.8% (65.4-88.2)	NS	NS
Gender (male)	55.5% (50.5-60.5)	57.9% (49.2-66.7)	54.3% (48.1-60.4)	NS	54.3% (42.3-66.3)	62.5% (49.4-75.6)	NS	NS
PD duration (years)	6.0 (5.6-6.5)	6.4 (5.7-7.2)	5.9 (5.3-6.4)	NS	6.8 (5.6-7.9)	6.0 (5.6-6.5)	NS	NS
Current smoker	8.2% (5.3-11.1)	8.7% (3.5-13.9)	8.0% (4.5-11.5)	NS	10.8% (3.0-18.5)	6.0% (0-12.8)	NS	NS
BMI	26.7 (26.3-27.1)	26.9 (26.2-27.5)	26.6 (26.0-27.1)	NS	26.8 (25.8-27.8)	27.0 (26.1-27.9)	NS	NS
Overweight (25≤BMI<30)	42.1% (37.2-47.1)	48.4% (39.6-57.3)	39.1% (33.2-45.1)	0.053	41.4% (29.6-53.3)	57.1% (43.8-70.5)	NS	NS
Obese BMI ≥ 30	18.5% (14.6-22.4)	17.5% (10.7-24.2)	19.9% (14.2-23.8)	NS	21.4% (11.6-31.3)	12.5% (3.6-21.4)	NS	NS
Diagnosed arterial hypertension	34.9% (30.1-39.7)	39.7% (31.0-48.3)	32.6% (26.8-38.3)	NS	31.4% (20.3-42.6)	50.0% (36.5-63.5)	0.03	NS
Diagnosed diabetes	11.7% (8.5-15.0)	13.5% (7.4-19.5)	10.9% (7.0-14.7)	NS	11.4% (3.8-19.1)	16.1% (6.1-26.0)	NS	NS
LED (mg)	646 (608-684)	706 (639-772)	617.1 (571-663)	0.02	707 (622-792)	704 (596-812)	NS	0.06
RBD	32.8% (28.1-37.5)	-	-	-	-	-	-	-
Sleep talking	23.7% (19.4-28.0)	55.6% (46.8-64.3)	8.1% (4.8-11.5)	<0.001	100%	-	0.0	0.0
Other sleep vocalizations	43.8% (38.8-48.7)	38.9% (30.3-47.5)	46.1% (40.0-52.2)	NS	-	87.5% (78.6-96.4)	0.0	0.0
Depression WHO-5 ≤ 28	13.3% (9.9-16.7)	19.8% (12.8-27.0)	10.1% (6.4-13.8)	0.007	17.1% (8.1-26.2)	23.2% (11.8-34.6)	NS	NS
Hallucinations (≥1/w)	14.3% (10.8-17.8)	26.1% (18.4-34.0)	8.5% (5.1-12.0)	<0.001	30.0% (19.0-41.0)	21.4% (10.3-32.5)	NS	0.0
Constipation (≥3/w)	29.2% (24.6-33.7)	46.0% (37.2-54.9)	20.9% (15.9-25.9)	<0.001	44.3% (32.3-56.2)	48.2% (34.7-61.7)	NS	0.002
Olfactory dysfunction	66.1% (61.3-70.8)	72.6% (64.6-80.5)	62.9% (56.9-68.8)	0.04	75.7% (65.4-86.0)	68.5% (55.7-81.3)	NS	0.04
ESS score	8.6 (8.1-9.1)	10.0 (9.1-10.8)	7.9 (7.3-8.5)	0.0	10.3 (9.0-11.6)	9.5 (8.3-10.7)	NS	0.002
ESS score > 10	33.1% (28.3-37.8)	41.3% (32.6-50.0)	29.1% (23.5-34.6)	0.01	47.1% (35.2-59.1)	33.9% (21.1-46.7)	NS	0.05

Sleep talkers had more non-motor symptoms compared to non-sleep talkers and a higher intake of PD medications (see Table 2). Age, gender, and PD duration did not differ between sleep talkers and non-sleep talkers.

**Table 2:** Characteristics of “non-sleep talkers” and “sleep talkers” Values are given as means or percentages, followed by a 95% confidence interval in parentheses, and median and IQR are given. The P-value is calculated for the difference between sleep talkers and non-sleep-talkers. Statistically significant differences are printed in bold. NS: Statistically not significant; y: Years; PD: Parkinson’s disease; LED: Levodopa equivalent dose; RBDSQ: REM sleep behavior disorder screening questionnaire; ESS: Epworth Sleepiness Scale

	Non-sleep talkers (N=293), mean (95% CI); median, IQR	Sleep talkers (N=91), mean (95% CI); median, IQR	P-value
Age (y)	67.3 (66.3-68.3); 66.4, 61.7-73.5	67.8 (65.8-69.8); 67.9, 61.8-75.1	NS
Gender, male	55.0% (49.2-60.7)	57.1% (46.8-67.5)	NS
PD duration (y)	5.8 (5.3-6.2); 5.4, 3.0-8.1	6.9 (5.8-8.1); 5.9, 3.0-9.7	NS
LED (mg)	621 (577-665); 560, 350-829	728 (653-803); 677, 460-958	0.006
RBDSQ score	3.6 (3.4-3.9); 3, 2-5	7.8 (7.3-8.3); 7, 6-10	<0.001
RBDSQ score ≥6	19.1% (14.6-23.6)	77.0% (68.1-85.7)	<0.001
Depression WHO-5 ≤ 28	12.6% (8.8-16.5)	15.4% (7.8-22.9)	NS
Hallucinations (≥ 1/w)	9.2% (5.9-12.5)	30.8% (21.1-40.4)	<0.001
Constipation	24.9% (19.9-30.0)	42.9% (32.5-53.2)	0.001
Olfactory dysfunction	63.4% (57.9-69.0)	74.4% (65.3-83.6)	0.055
ESS score	8.1 (7.6-8.7); 8, 4-11	10.0 (8.9-11.2); 10, 6-14	0.002
ESS score > 10	28.7% (23.5-33.9)	47.3% (36.8-57.7)	0.001

Table 3 displays the results of the mortality models. REM sleep behavior disorder (RBD, defined as RBDSQ ≥6) was not significantly associated with increased mortality (HR: 1.43; 95% CI: 0.92-2.22) in univariate models. In univariate models, RBD (RBDSQ ≥6) with ST was associated with increased mortality (HR: 1.90; 95% CI: 1.18-3.06), and RBD without ST was not (HR: 0.77; 95% CI: 0.40-1.50).

**Table 3:** Hazard ratios for mortality in Parkinson’s disease (N=384) Values are given as hazard ratios, followed by a 95% confidence interval in parentheses. Statistically significant differences are printed in bold. y: Years; BMI: Body mass index; PD: Parkinson’s disease; LED: Levodopa equivalent dose; RBD: REM sleep behavior disorder

	Univariate model	Model 1	Model 2	Full model
Age (y)	1.07 (1.04-1.09)	1.05 (1.02-1.08)	1.05 (1.03-1.08)	1.05 (1.02-1.08)
Gender, male	1.26 (0.81-1.96)	1.31 (0.84-2.07)	-	1.30 (0.82-2.07)
BMI	0.93 (0.87-0.98)	0.91 (0.86-0.97)	0.91 (0.86-0.97)	0.92 (0.86-0.97)
PD duration (y)	1.09 (1.04-1.14)	1.05 (1.00-1.10)	1.06 (1.01-1.10)	1.05 (1.00-1.11)
Diagnosed arterial hypertension	1.54 (1.00-2.39)	1.88 (1.17-3.0)	1.83 (1.15-2.90)	1.89 (1.18-3.03)
LED (mg)	1.00 (1.00-1.00)	-	-	1.00 (0.99-1.00)
RBD	1.43 (0.92-2.22)	0.79 (0.46-1.35)	-	0.79 (0.46-1.37)
Sleep talking	2.17 (1.39-3.38)	2.38 (1.39-4.07)	2.08 (1.30-3.29)	2.22 (1.10-4.51)
Sleep vocalization	0.70 (0.45-1.10)	-	-	0.97 (0.54-2.89)
Depression	1.86 (1.09-3.17)	1.66 (0.93-2.97)	-	1.60 (0.89-2.89)
Hallucinations (≥1/w)	1.65 (0.97-2.81)	-	-	1.21 (0.68-2.17)

We tested associations between separate RBDSQ questions and mortality. Only three questions from the RBDSQ showed statistical significance about mortality: question 4 on limb movement during sleep (HR: 1.30; 95% CI: 1.13-3.49); question 6.1 on speaking, shouting, swearing, or laughing loudly during dreams (HR: 2.43; 95% CI: 1.43-4.13); and question 6.2 about sudden limb movements or “fighting” in sleep (HR: 1.66; 95% CI: 1.05-2.63).

Other sleep-talking conditions were tested in a separate model. After adjusting for possible traits that are associated with ST (i.e., RBD, insomnia, sleepwalking as non-rem parasomnia, and depression), ST was still significantly associated with mortality (HR: 2.42; 95% CI: 1.35-4.34). Sleepwalking was reported by eight participants. Insomnia or sleepwalking was not associated with increased mortality in univariate models (insomnia: HR: 1.04; 95% CI: 0.68-1.61 and sleepwalking: HR: 1.08; 95% CI: 0.78-1.50).

**Figure 1:**
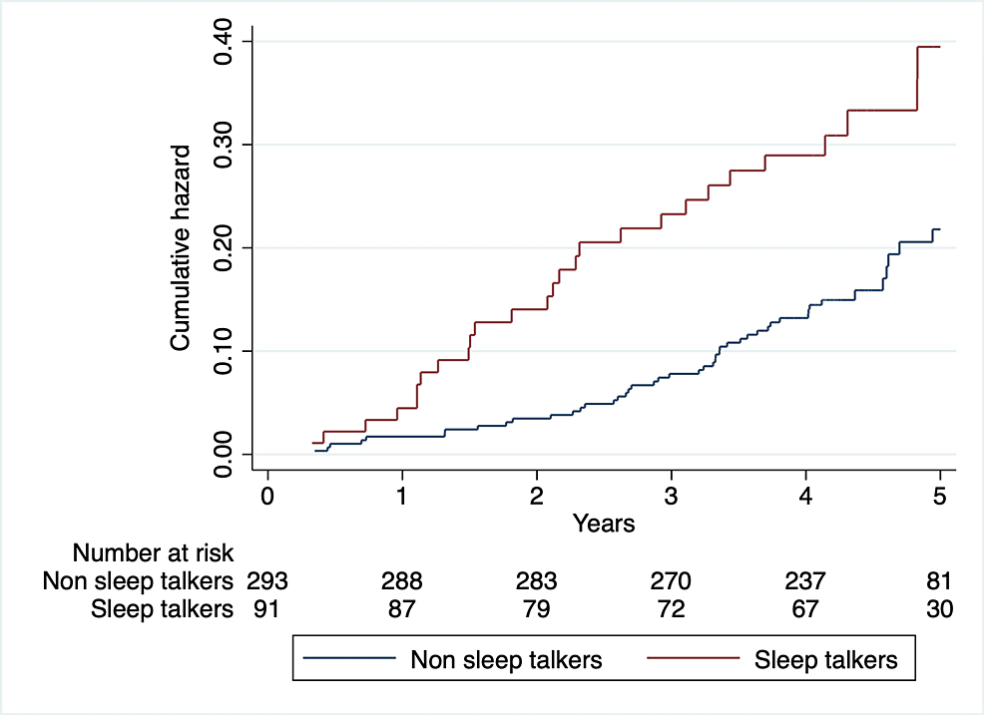
Nelson-Aalen estimates of cumulative mortality for Parkinson’s disease patients with and without sleep talking

Weekly or more frequent ST was associated with increased mortality in all tested models. There was no statistically significant interaction between ST and RBD (HR: 1.20; 95% CI: 0.39-3.70). The Nelson-Aalen cumulative hazard estimates for ST are shown in Figure 1.

## Discussion

The best-known prognostic factors associated with early mortality in PD are male gender, older age at diagnosis, more severe PD at baseline, and cognitive impairment [12]. The use of antipsychotic medications is also associated with higher early mortality in PD [13]. As far as we know, this is the first long-term follow-up study on the effect of ST on mortality in PD. Our new findings suggest that frequent ST (talking in their sleep at least one night a week) is a new risk factor for mortality in PD. RBD with ST was associated with increased mortality, while RBD without ST was not. ST has been previously regarded only as a benign isolated symptom. ST seems to be more common in Lewy body dementia compared to Alzheimer’s disease, vascular dementia, and frontotemporal lobar degeneration [4]. In our study, we found frequent ST to be an independent risk factor for mortality.

RBD is considered a prodromal manifestation of alpha-synucleinopathies, including Lewy body disease, PD, and multiple system atrophy [14]. Eventually, more than 80% of patients with RBD develop PD or dementia [15,16]. Diagnosis is based on polysomnography, where decreased REM sleep atonia is the main finding. Clinically, RBD is accompanied by abrupt movements, vocalizations, and dream-related symptoms, such as frightening and emotional dreams and frequent nightmares. RBD in PD has been linked to a higher risk of developing cognitive impairment [5]. In cross-sectional and longitudinal studies, idiopathic RBD is associated with increased cognitive impairment [17], although some controversial reports exist [18]. The link between RBD and more rapid cognitive decline does not manifest as increased mortality in our study, RBD without ST was not associated with increased mortality.

ST is a common phenomenon that can occur in both non-REM and REM sleep. ST is often present in RBD. The questionnaires on RBD, including the Marburg questionnaire that we used [9], include ST as one symptom of RBD. It remains unclear whether people with frequent ST are more prone to develop PD than people who do not talk in their sleep. This should be studied further.

Our data show that weekly or more frequent ST is associated with increased mortality. Association with increased mortality is not seen when ST is present less than once a week. If ST is only screened for by asking whether or not it occurs (yes/ no), the frequency of the symptom cannot be established. According to our results, occasional ST is normal, but frequent (talking in their sleep at least once a week) is associated with higher mortality in PD patients. Other contributing factors to this finding could be, for example, the definition of RBD in our study. Different RBD phenotypes may have higher mortality. ST per se could also be a marker for PD progression or neurodegeneration. ST could also be more prevalent with multiple system atrophy or dementia with Lewy bodies.

Confounding factors could explain part of this. In our study, sleep talkers reported more hallucinations. The prevalence of hallucinations in our study is similar to those of previous studies [19]. Sleep talkers are sleepier and have a higher prevalence of constipation when compared to non-sleep talkers. A similar trend is seen with olfactory dysfunction. These differences between sleep talkers and non-sleep talkers could explain mortality differences to some extent. Non-motor symptoms have been previously associated with increased mortality. Other conditions that are associated with ST were tested for separately, including RBD, non-REM parasomnias, insomnia, and depression. Our findings suggest that ST is an independent risk factor for mortality, even after adjusting for age, gender, disease duration, medication, depression, hallucinations, BMI, and RBD. Unfortunately, we do not have information about possible anxiety, medications (antidepressants, somnotics, and antipsychotics), or drug use that could also affect ST and explain the results.

RBDSQ was used as a validated marker of RBD [9]. We are not aware of studies about associations between RBDSQ and mortality. The validity of RBDSQ (total score) has been tested both in healthy subjects, idiopathic RBD, different sleep disorders, and PD [10]. In PD when using a cutoff score of 6 points, the sensitivity and specificity were 84.2% and 96.2%, respectively [10].

Why was at least weekly ST associated with mortality but a total score of RBDSQ was not? This discrepancy is difficult to explain. ST could represent clinical RBD and the severity of RBD better than the RBDSQ score. Other conditions that could affect ST were also tested (i.e., depression, other non-REM parasomnias (sleepwalking), and hallucinations), but these did not change the results. Study protocol may have a role too. Recall bias may explain part of this. Subjects living alone are probably unaware of the presence of RBD symptoms, but this is also true for ST. Another problem with RBDSQ is that it lacks quantitative information about symptoms. RBDSQ pools information about laughing, swearing, and shouting during dreams together with ST. RBD was associated with mortality when combined with ST and RBD without ST was not. This too hints at the importance of ST. We need more studies to understand this.

We can only speculate what possible biological processes could explain the association between ST and increased mortality in PD. The pathophysiology behind ST is not well-known. The abundance of sleep disturbance and neuropsychiatric symptoms in PD could hint toward shared mechanisms. We think that ST could be a sign of an ongoing neurodegeneration similar to “acting out in dreams” in RBD. We also know that disturbances of the autonomic nervous system are essential elements of PD, and, in our study, ST is associated with non-motor symptoms, as seen in Table 2. Sleep architecture is also affected in PD, and this could lead to increased ST [20]. REM sleep without muscle atonia is also a hallmark of RBD. Therefore, it is also possible that someone talks while dreaming during a nocturnal RBD attack. The lack of muscle inhibition probably increases the probability of speaking aloud while dreaming.

The prevalence of ST in a normal elderly population is not well-known. In an adult population with a mean age of 47 years (ranging from 18 to 96 years), the prevalence of at least weekly ST was 6.3% [21]. In a study with 317 elderly patients with different dementias, and a control group of 32 non-demented elderly, the prevalence of frequent ST in the non-demented elderly was 6.3%. The prevalence of frequent ST in patients with Lewy body dementia was 36.4%. In this study, the threshold for frequent ST was once a week (i.e., similar to our study) [4].

ST can involve complicated dialogues or monologues, complete gibberish, or mumbling. It can differ in frequency and volume from person to person. We did not differentiate between different types of ST, but usually, it is understood as talking, instead of mumbling or shouting.

There is limited data on the effect of RBD on mortality in PD [22]. Our finding, concerning RBD, is in line with a previous questionnaire study, where probable RBD was estimated using a part of the Stavanger Sleepiness Questionnaire [3]. Contrary to our finding, PSG confirmed RBD has previously been shown to increase mortality in patients with a neurodegenerative disease, although the type of neurodegenerative disease was not specified [23].

We investigated the relationship between PD, hypertension, and body weight. Arterial hypertension is a well-known risk factor for cardiovascular mortality. Similarly, loss of weight and a decrease in BMI is associated with frailty in elderly people. Although severe obesity is a risk factor for mortality, moderate overweight may be a protective factor among elderly people [24] and in patients with neurodegenerative disease. In a large Swedish study, a reduction in mortality was seen with increased BMI up to 29.9 kg/m^2^ for men and 24.9 kg/m^2^ for women [25]. There is also evidence that weight loss may worsen the prognosis for PD patients [26]. Our study results support the evidence that treating hypertension should be taken seriously and that a reduction in body weight in PD patients is not advantageous.

There was a lack of a clear relationship between dopaminergic treatment and mortality in our study. It is well-known that LED reflects the development of motor symptoms [8], but the effect of medications on mortality is not well-established. Similar to previous results, we found no association between LED and mortality [27]. The duration of disease was significantly associated with increased mortality in the univariate model, but not in adjusted models.

The strengths of our study are the large sample size and well-represented follow-up information. The response rate was high for a questionnaire study, which is typical for a Finnish questionnaire study. This could be partly driven by the type of survey (mail questionnaire) and the high motivation of subjects as they were registered members of the Finnish Parkinson’s Association. Subjects were randomly selected from members of the Finnish Parkinson’s Association. Patients usually become members of the association some years after a PD diagnosis has been confirmed, but some leave the association in an advanced state of the disease. Only a minority of patients were living in elderly homes, but this is in line with Finnish institutional care statistics. The size of the follow-up population is acceptable, as patients with partially answered questionnaires were not included in the study. Otherwise, only 39 patients were lost to follow-up. The reasons for loss to follow-up included a change of name, moving abroad, or no address information or telephone number could be found in the address registers. Our study sample is, thus, a representative sample of “middle-stage” Finnish Parkinson patients, as has been reported before [6].

Our study has several limitations. Clinical diagnosis of PD is a challenge. All patients included in the study have been diagnosed with PD by a neurologist. Nevertheless, it is possible that the definite diagnosis (based on pathomorphological assessment) would be different in some cases. Our study population may include some subjects with different neurodegenerative diseases, for example, dementia with Lewy bodies, and multiple system atrophy, in addition to PD. Patients with significant cognitive decline are probably underrepresented in our study population. It is also possible that non-responders had a more rapid disease phenotype or higher mortality compared to the responders. Unfortunately, we also collected insufficient data to be able to evaluate dementia. Because of limited funding resources, we were unable to meet all patients personally. Because of the lack of clinical neurological examinations, we could not estimate the progression and stage of PD objectively.

There is a possibility of recall bias. Subjects may not be aware of their ST or symptoms of RBD if they are living alone. Both ST and RBD symptoms are probably reported more often by subjects who are living together with someone. We asked responders to ask other persons about their symptoms if they were not aware of their ST or other symptoms. Even then, it is possible that some cases of frequent ST have not been detected. It is unlikely that frequent ST has been reported if that is not true. To estimate ST, we used quantitative phrasing as in the BNSQ, which allows better comparability between subjects than purely qualitative questions [7]. It is still a possibility that some responders were talking in their sleep at least weekly, even if they were reported as not talking in their sleep. In our study, we defined “sleep talkers” to be only those who reported talking in their sleep at least once per week similar to that of Honda et al. [4]. To measure ST objectively, we would need night-to-night sleep recordings combined with analysis of sounds to differentiate ST, for example, from snoring and other sounds. Unfortunately, we lacked the resources for such monitoring. We had to rely on the history given by the sleepers and their bed partners.

The best way to confirm RBD is to perform video polysomnography (PSG). Unfortunately, we were unable to perform PSGs due to lack of funding. However, this should not be seen as a major shortcoming, as we aimed to gather information about sleep and PD, not only RBD. As mentioned, a PSG would be insufficient when determining the prevalence of ST. A single night PSG may reveal REM sleep without atonia, and dream-related behavior may be detected in a video PSG. However, the weekly to monthly frequency cannot be determined with one-night PSG. We used the RBD questionnaire and asked about the frequency of ST and the frequency of RBD-like behavior, as described earlier [6]. In this way, asking about the occurrence and frequency of ST is easier, and it seems to provide some information on the prognosis of PD. So far, we do not know if frequent ST is a risk factor for developing PD in the same way as RBD.

Our results are important for various reasons. History of ST should be regarded as a clinically significant symptom when assessing the risk of mortality in PD, or when diagnosing RBD. A clinician can easily ask about ST and its frequency. The scale in our study is the same as that in the BNSQ [7].

Based on our study, strong inferences about the causality between frequent ST causes of mortality cannot be made. Additionally, we cannot make any inferences about frequent ST as a risk factor for developing PD. Further studies are needed to distinguish between different types of ST. It is relatively easy to screen for and measure ST with new technology and portable devices; thus, these could be used for more precise phenotyping. It would be interesting to know if ST could be used to distinguish PD and other neurodegenerative disorders from drug-induced extrapyramidal symptoms or essential tremors. We studied ST in patients diagnosed with PD. It is, nevertheless, possible that ST is a prodromal symptom of both PD and idiopathic RBD. If that is the case, it would mean that frequent ST is not just a benign symptom. Conversely, occasional ST (talking while asleep, say, once a month or more rarely) probably lacks clinical significance.

## Conclusions

According to our study, frequent sleep talking (talking while asleep at least one night per week) is a risk factor for early mortality in PD. RBD with sleep talking was associated with increased mortality, while RBD without sleep talking was not. Other important risk factors included the age of the subjects at entry, duration of PD, arterial hypertension, and body mass index. A higher body mass index was a protective factor, whereas a higher age and longer duration of PD were risk factors. The presence of RBD, gender, depression, and amount of PD medication was not significantly associated with mortality. Frequent sleep talking could also be an indicator of more severe PD. Occasional sleep talking, on the contrary, is an isolated symptom. Therefore, asking PD patients about frequent sleep talking may have important clinical implications. Future prospective studies are needed.
